# Functional Mapping of Transcription Factor Grf10 That Regulates Adenine-Responsive and Filamentation Genes in Candida albicans

**DOI:** 10.1128/mSphere.00467-18

**Published:** 2018-10-24

**Authors:** Tanaporn Wangsanut, Joshua M. Tobin, Ronda J. Rolfes

**Affiliations:** a Department of Biology, Georgetown University, Washington, DC, USA; University of Texas Health Science Center

**Keywords:** *Candida albicans*, morphogenesis, purine metabolism, transcription factors

## Abstract

Grf10, a homeodomain-containing transcription factor, regulates adenylate and one-carbon metabolism and morphogenesis in the human fungal pathogen Candida albicans. Here, we identified functional domains and key residues involved in transcription factor activity using one-hybrid and mutational analyses. We localized activation domains to the C-terminal half of the Grf10 protein by one-hybrid analysis and identified motifs using bioinformatic analyses; one of the characterized activation domains (AD1) responded to temperature. The LexA-Grf10 fusion protein activated the *lexA_op_-HIS1* reporter in an adenine-dependent fashion, and this activation was independent of Bas1, showing that the adenine limitation signal is transmitted directly to Grf10. Overexpression of LexA-Grf10 led to filamentation, and this required a functioning homeodomain, consistent with Grf10 controlling the expression of key filamentation genes; filamentation induced by LexA-Grf10 overexpression was independent of adenine levels and Bas1. Alanine substitutions were made within the conserved interaction regions (IR) of LexA-Grf10 and Grf10 to investigate roles in transcription. In LexA-Grf10, the D302A mutation activated transcription constitutively, and the E305A mutation was regulated by adenine. When these mutations were introduced into the native gene locus, the D302A mutation was unable to complement the ADE phenotype and did not promote filamentation under hypha-inducing conditions; the E305A mutant behaved as the native gene with respect to the ADE phenotype and was partially defective in inducing hyphae. These results demonstrate allele-specific responses with respect to the different phenotypes, consistent with perturbations in the ability of Grf10 to interact with multiple partner proteins.

**IMPORTANCE** Metabolic adaptation and morphogenesis are essential for Candida albicans, a major human fungal pathogen, to survive and infect diverse body sites in the mammalian host. C. albicans utilizes transcription factors to tightly control the transcription of metabolic genes and morphogenesis genes. Grf10, a critical homeodomain transcription factor, controls purine and one-carbon metabolism in response to adenine limitation, and Grf10 is necessary for the yeast-to-hypha morphological switching, a known virulence factor. Here, we carried out one-hybrid and mutational analyses to identify functional domains of Grf10. Our results show that Grf10 separately regulates metabolic and morphogenesis genes, and it contains a conserved protein domain for protein partner interaction, allowing Grf10 to control the transcription of multiple distinct pathways. Our findings contribute significantly to understanding the role and mechanism of transcription factors that control multiple pathogenic traits in C. albicans.

## Abstract (precis)

Metabolic adaptation and morphogenesis are essential for Candida albicans, a major human fungal pathogen, to survive and infect diverse body sites in the mammalian host. C. albicans utilizes transcription factors to tightly control the transcription of metabolic genes and morphogenesis genes. Grf10, a critical homeodomain transcription factor, controls purine and one-carbon metabolism in response to adenine limitation, and Grf10 is necessary for the yeast-to-hypha morphological switching, a known virulence factor. Here, we carried out one-hybrid and mutational analyses to identify functional domains of Grf10. Our results show that Grf10 separately regulates metabolic and morphogenesis genes, and it contains a conserved protein domain for protein partner interaction, allowing Grf10 to control the transcription of multiple distinct pathways. Our findings contribute significantly to understanding the role and mechanism of transcription factors that control multiple pathogenic traits in C. albicans.

## INTRODUCTION

The ability of organisms to sense changes in environmental conditions and respond to them is essential for viability. Transcriptional regulators control gene expression to orchestrate responses to different signaling cues and to maintain homeostasis in continually fluctuating environments. Candida albicans is a commensal fungus that resides naturally in the human gastrointestinal tract, in mucosal membranes, and on the skin (1, 2). However, C. albicans can also cause superficial yeast infections in immunocompetent people and lethal systemic infections in those with weakened immune systems (3–5). As a result, C. albicans has a remarkable ability to adapt itself to the changing physiological conditions in human hosts.

Transcriptional control plays a central role in the regulation of pathogenic-related attributes that are mainly involved in metabolic fitness and virulence (4, 6). Fitness attributes are required to support cellular growth and survival, while virulence attributes are required to increase the likelihood of C. albicans to cause and establish infections. Transcription regulators, such as Efg1, Tup1, Gcn4, and Ace2, have been showed by transcriptomic analyses to regulate the expression of metabolic and virulence genes in C. albicans (7–10). These studies underscore the critical role of transcription factors in coordinating the expression of fitness- and virulence-related genes for C. albicans to respond and survive in changing environments.

Recently, we established the role of the homeodomain-containing transcription factor Grf10 as an additional regulator that controls metabolism and virulence in C. albicans (11, 12). Grf10 in conjunction with the transcription factor Bas1 regulates metabolism by upregulating the expression of genes for adenylate biosynthesis (*ADE* genes), one-carbon metabolism, and a nucleoside permease (*NUP*) under adenine limitation (12). Grf10 is implicated in virulence attributes by controlling yeast-hypha morphogenesis (11), one of the most well-documented virulence factors found in C. albicans (13). The *grf10*Δ mutant exhibits hyphal growth defects and has attenuated virulence in animal models of infection (11, 14). Overexpression of *GRF10* triggers hyphal formation under yeast-promoting conditions, and the expression of the *GRF10* gene is upregulated under hypha-inducing conditions (11, 15–17). Together, these results emphasize a critical role for the Grf10 transcription factor in governing C. albicans growth and virulence.

Even though several studies have recognized that transcription factors coregulate metabolism and virulence in C. albicans, insights into the mechanisms for this integration are still missing. In this study, we mapped the functional domains of Grf10. Using artificial constructs containing the LexA DNA-binding domain and the *lexA_op_-HIS1* reporter system (18), we mapped two activation domains toward the C terminus of Grf10, one of which responds to temperature, and found that LexA-Grf10 directly senses the adenine limitation signal without the coregulator Bas1. Overexpression of LexA-Grf10 drove filamentation; the homeodomain, but not Bas1 or adenine levels, was required for LexA-Grf10 to induce filamentation. A conserved interaction region (IR) is predicted to mediate interactions with protein partners necessary for transcription. Mutation of residues within the IR, D302A and E305A, and introduction of the mutant gene into strains led to a weak adenine auxotrophy, an inability to promote hyphal growth in the native Grf10 protein, and unregulated activation in the LexA-Grf10 protein. Together, our results provide evidence for separate regulation of adenylate biosynthesis and filamentation by Grf10 and suggest that intramolecular interactions mask activation domains until interrupted by protein partner interactions.

## RESULTS

### Functional mapping of Grf10 activation domains.

**FIG 1:**
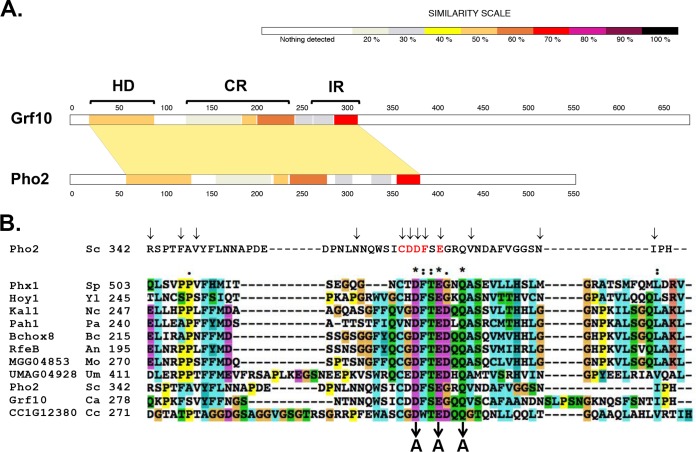
Protein alignment of C. albicans Grf10 and fungal orthologues reveals conserved regions. (A) C. albicans Grf10 was aligned with S. cerevisiae Pho2. The aligned region, shaded in tan, has an overall identity of 40.1% in 334 amino acids; the amino and carboxy termini do not align. The degree of sequence conservation is indicated by the scale at the top. The brackets indicate the regions of Grf10 defined based on the corresponding functional domains from ScPho2, as follows: HD, DNA-binding homeodomain; CR, central region; IR, interaction region. (B) Conservation of the IR in multiple fungal species. Top line is the sequence of the IR from ScPho2; arrows indicate positions of point mutations that inhibit interactions with its three coregulators, and red letters indicate the core of the Pho2 interaction region (Bhoite et al. [22]). Below, alignment of the IR from multiple fungal species. Sp, Schizosaccharomyces pombe; Yl, Yarrowia lipolytica; Nc, Neurospora crassa; Pa, *Podospora anserine*; Bc; *Botrytis cinerea*; An, Aspergillus nidulans; Mo, Magnaporthe oryzae; Um, *Ustilago maydis*; Sc, Saccharomyces cerevisiae; Ca, Candida albicans; and Cc, *Coprinopsis cinerea*. The phylogenetic tree from this analysis is found in Fig. S1. The number indicates the position of the first listed amino acid. Protein sequence alignment was performed using ClustalX2.1. Amino acids are highlighted based on their biochemical characteristics and conservation: blue, hydrophobic; magenta, acidic; green, polar; orange, glycine; yellow, proline; cyan, aromatic. Symbols above the alignment indicate the conservation: asterisk (*) indicates a single fully conserved amino acid, colon (:) indicates amino acid groups with strongly similar properties, and dot (·) indicates groups with weakly similar properties.

We reasoned that understanding the structure-function relationship of the Grf10 protein might lead to insights into how Grf10 regulates adenylate metabolism and filamentation. We compared Grf10 with *Sc*Pho2 (*Sc*Pho2 is the Saccharomyces cerevisiae orthologue of Grf10) and with other Candida species (19) using BLAST and SIM tools. As shown in Fig. 1A, Grf10 is composed of 685 amino acids and contains a highly conserved DNA-binding homeodomain near the N terminus (amino acids 38 to 96). There are two additional conserved regions: the first region, the central region (CR), is located between amino acids 154 and 248 and is conserved among hemiascomycetes, species that are closely related to Candida spp. (19, 20); the second region is the interaction region (IR), is located between amino acids 272 and 351, and contains residues that are conserved across a wider range of fungal species, including both ascomycetes and basidiomycetes (Fig. 1B; see also Fig. S1 in the supplemental material). Interestingly, these proteins are involved in morphological development and nutrient metabolism (Table 1). In S. cerevisiae**,** the IR is required for protein partner interactions (21, 22). The carboxy-terminal half of the protein lacks conserved sequences or identified functional domains. Because Grf10 promotes gene expression (12), we hypothesized that it contains activation domain(s), as opposed to transcriptional repression domains; however, unlike other functional domains, the sequences of activation domains are poorly conserved and are defined experimentally for individual transcription factors (23).

**TABLE 1:** Fungal homeodomain proteins with a conserved interaction region

Phylum	Species	Description	Gene(s)	Phenotype	Reference(s)
Ascomycota	Candida albicans	Dimorphic yeast, human commensal and opportunistic pathogen	*GRF10*	*grf10*Δ shows filamentation defect; overexpressed *GRF10* enhances filamentous growth; *GRF10* is upregulated during biofilm development; Grf10 is required for transcription of genes in response to adenine starvation	11, 12, 15, 16
	Saccharomyces cerevisiae	Budding yeast, nonpathogen	*PHO2*	*pho2*Δ has sporulation defect and abnormal bud morphology; Pho2 regulates genes in response to phosphate and adenine starvation; Pho2 activates gene involved in initiation of mating type switching during vegetative cell division	22, 60, 61
	Schizosaccharomyces pombe	Fission yeast, nonpathogen	*phx1^*+*^*	Phx1 regulates long-term survival under nutrient starvation; *phx1*Δ shows meiotic sporulation defect; Phx1 regulates transcription of thiamine responsive genes	62, 63
	Neurospora crassa	Filamentous fungus, nonpathogen	*Kal-1*	*Kal-1*Δ exhibits delayed basal hyphal growth, defective conidiation; Kal-1 may have a role in nutrient sensing	64
	Podospora anserine	Filamentous fungus, nonpathogen	*Pah1*	*Pah1* plays roles in hyphal morphogenesis and development of female ascogonia (microconidiogenesis)	65
	Botrytis cinerea	Plant pathogen	*BcHOX8*	*Bchox8*Δ exhibits a deformed morphology (arabesque), decreased conidial production, and attenuated virulence	66
	Yarrowia lipolytica	Dimorphic yeast	*HOY1*	*hoy1*Δ abolishes hyphal growth, while overexpressed *HOY1* enhances filamentation	67
	Magnaporthe oryzae	Rice blast fungus, plant pathogen	*MoHOX2 (HTF1), MoHOX7*	*MoHOX2* is required for conidiogenesis but not for appressorium-related pathogenic development; *Mohox2*Δ exhibits normal hyphal growth; *MoHOX7* is required for normal appressoria formation	68, 69
	Aspergillus nidulans	Filamentous fungus, nonpathogen	*RfeB*	*RfeB* plays roles in asexual development, including hyphal growth and conidiation	70
Basidiomycota	Ustilago maydis	Corn smut, plant pathogen	*UMAG04928*	Hypothetical protein, uncharacterized phenotype	71
	Coprinopsis cinerea	Multicellular fungus, edible mushroom	*CC1G_12380*	Hypothetical protein, uncharacterized phenotype	72

10.1128/mSphere.00467-18.4 FIG S1 Phylogenetic tree of fungal IR residues from Fig. 1B. The tree was generated using ClustalX 2.1 (N-J tree with 1,000 bootstraps) and was viewed in MEGA7 (rooted by midpoint). Download FIG S1, PDF file, 0.05 MB. Copyright © 2018 Wangsanut et al. 2018 Wangsanut et al. This content is distributed under the terms of the Creative Commons Attribution 4.0 International license.

**FIG 2:**
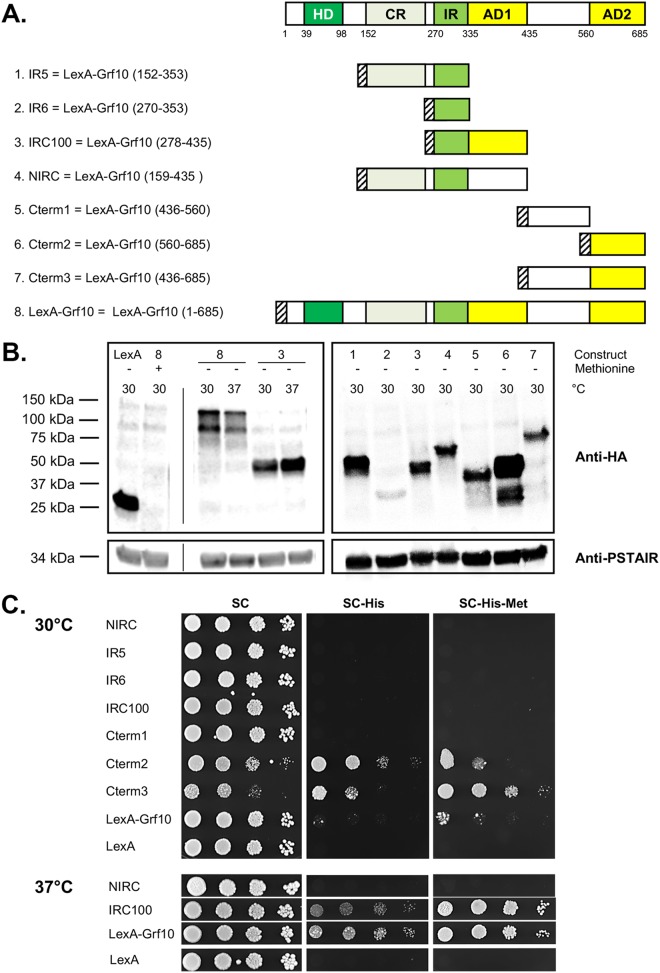
Grf10 contains two activation domains. (A) Schematic (top) depicts the conserved regions (in green, see Fig. 1) and newly mapped functional domains (in yellow) of Grf10; numbers below the box indicate the amino acid number. Below, schematic representations of the Grf10 fragments cloned into pC2HB. LexA-HA is depicted as a striped box. AD1 and AD2, activation domain 1 and 2, respectively. (B) Immunoblot assay to determine protein stability. Left, LexA is the empty vector; 8 and 3 indicate the full-length LexA-Grf10 and IRC100 constructs from panel A, respectively. The presence or absence of methionine in the SC growth medium is represented by + or −, respectively, and the temperature for growth of the strains is indicated by 30 for 30°C or 37 for 37°C. The thin vertical line indicates position of lanes deleted by Photoshop to allow juxtaposition of control and experimental lanes. Experiments were performed twice, and band intensities were quantified using the ImageJ software, normalized to PSTAIRE, and averaged; a representative immunoblot is shown. Right, lanes marked 1 to 7 correspond to the same numbers in panel A to indicate the LexA fusions with portions of Grf10. Anti-HA (top) detects the LexA-Grf10 fusion proteins, and the anti-PSTAIR (bottom) was used to detect the cyclin-dependent kinase Cdc28 as a protein loading control. (C) Growth on medium lacking histidine to detect expression of the *HIS1* reporter gene. Strains were grown overnight in YPD medium, diluted in sterile water (see Materials and Methods), serially diluted 1:10, and spotted on SC medium (containing histidine) or on SC-His medium with or without methionine, as indicated; the absence of methionine induces expression of LexA proteins. The plates were incubated at 30°C (top) or at 37°C (bottom) and photographed at 48 h. Representative plates are shown; at least three transformants of each construct were assayed.

To map the activation domain(s) of Grf10, we fused various portions as well as the full-length *GRF10* gene to the CUG codon-optimized LexA DNA-binding domain (18), as shown in Fig. 2A. Our choices for *GRF10* fragments came from assessing regions of conservation obtained from protein alignments between Grf10 and other fungal homeodomain-containing proteins. The fusion protein constructs were expressed under the inducible *MET3* promoter, which drives construct overexpression under methionine limitation conditions and is moderately repressed under standard synthetic complete (SC) medium, which contains methionine (18, 24). Plasmids were transformed into the C. albicans strain harboring the *lexA_op_-HIS1* reporter. We confirmed that expression of Grf10 fusion proteins is inducible under methionine limitation (Fig. 2B, left; shown for the Grf10 full-length construct [data not shown for the truncation constructs]). The fusion proteins are all stable, as assessed by Western blot analysis, except for construct IR6 that carries only the IR (Fig. 2B, right).

We used one-hybrid assays to examine the ability of the Grf10 fragments to express *HIS1* by plating cells on SC-His medium. The Grf10 activation domains were localized to two regions (Fig. 2A). Activation domain 1 (AD1) was found between residues 278 and 435 in construct IRC100, which contains the conserved IR plus 100 amino acids next to it in the C-terminal direction (see Fig. 2C, bottom). Interestingly, AD1 activated the *HIS1* reporter gene at 37°C but not at 30°C (Fig. 2C, compare top and bottom). The full-length Grf10 fusion protein also activated the *HIS1* reporter at higher levels at 37°C than at 30°C (Fig. 2C, compare top and bottom), which may be due to AD1. Western analysis indicated little difference (less than 2-fold) in the stability of these fusion proteins at the two temperatures (Fig. 2B, left); steady-state levels of the full-length Grf10 at 37°C were ∼75% of those at 30°C, whereas the steady-state levels IRC100 at 37°C were 158% of those at 30°C. These small differences do not correlate with the growth of the strains on media lacking histidine. *In silico* analysis revealed a glutamine-rich (Q) region (13 of 24 amino acids) found from amino acids 353 to 376 within the AD1 region; glutamine-rich regions are known to be important for protein-protein interactions and transcriptional activation (25, 26) (Fig. S2A and B). Analysis also identified a nine-amino-acid transactivation domain (9aaTAD) motif (27) located from amino acids 345 to 353 within AD1 (QYLSQFILQ) (Fig. S2A and C). Both of these motifs are consistent with the idea that the region from amino acids 278 to 435 of the Grf10 functions as a transcriptional activation domain. Interestingly, a larger fragment, NIRC (amino acids 159 to 435) did not promote expression of the *HIS1* reporter at either temperature, suggesting that CR inhibits AD1 (see Fig. 2C, bottom).

10.1128/mSphere.00467-18.5 FIG S2 Computational analysis to identify sequence motifs found within the Grf10 activation domains. (A) Amino acid sequence of Grf10, retrieved from http://candidagenome.org. Bold amino acids indicate the experimentally identified Grf10 activation domains, AD1 from amino acids 278 to 435 and AD2 from amino acids 560 to 685. Amino acids highlighted in blue indicate the glutamine-rich region identified by Motif Scan, amino acids highlighted in purple indicate a short polyglutamine sequence identified by eye, and amino acids highlighted in red represent the nine-amino-acid transactivation domain (9aaTAD). Amino acids highlighted in green are outside the regions that showed the ability to active transcription and are represent a perfect match for the 9aaTAD motif. (B) Output of the web-based tool Motif Scan (http://myhits.isb-sib.ch/cgi-bin/motif_scan), using the PROSITE profiles as the motif source. (C) Output from the 9aaTAD prediction (http://www.med.muni.cz/9aaTAD/index.php) tool, using the moderate stringency criteria. Arrows indicate the 9aaTAD motifs found in the AD1 and AD2 regions. Download FIG S2, PDF file, 0.1 MB. Copyright © 2018 Wangsanut et al. 2018 Wangsanut et al. This content is distributed under the terms of the Creative Commons Attribution 4.0 International license.

The second activation domain (AD2) was found in constructs Cterm-2 and Cterm-3 (Fig. 2A), mapping it to the C terminus between amino acids 560 and 685, and AD2 affected growth differently from AD1. Cells were able to grow on medium lacking histidine when these two constructs were expressed at moderate or induced levels (on SC-His or SC-His-Met medium, respectively). However, cells exhibited attenuated growth on SC medium; this growth inhibition is not due to *HIS1* reporter gene expression because histidine was present. These findings are consistent with toxicity due to overexpression of an unregulated activation domain (28–30). The C-terminal region is 126 amino acids long (18% of the total length), and it carries several motifs associated with activation domains, as follows: (i) it is acidic (31% of the aspartic and glutamic acids and only 8% of the lysines and arginines) and contains 25% of the phenylalanines, consistent with the activation domains that are acidic with bulky hydrophobic residues (31), (ii) a 9aaTAD sequence is found from amino acids 675 to 683 (TNLDSFIDF) (Fig. S2A and C), and (iii) a short polyglutamine sequence is found from amino acids 663 to 666. Overall, we mapped the two activation domains in Grf10; both are located in the C-terminal half of the Grf10 protein.

### Interaction region of Grf10.

Grf10 regulates the expression of purine biosynthesis, one-carbon metabolism, and filamentation genes in C. albicans, and protein partner interaction may be necessary for regulation. The conservation of the interaction region paired with the characterized functional role in the *Sc*Pho2 protein led us to hypothesize that the IR is a Grf10 interaction domain. To map the Grf10 interaction domain, we used the Candida two-hybrid assay (18), repeating the positive and negative controls (Fig. S3A). Because Grf10 and Bas1 are both required to upregulate adenine-responsive genes (12), we used Grf10 fragments as “bait” and full-length Bas1 fused to VP16 as the “prey.” The minimal IR fragment, construct IR6, was unstable (Fig. 2B). We lengthened it by extending the IR in constructs IR5, IRC100, and NIRC; each of these constructs produced stable proteins (Fig. 2B). However, we could not detect an interaction between the LexA-fusion proteins IR5, IRC100, or NIRC and Bas1-VP16 (Fig. S3B). We flipped the constructs, placing full-length Grf10-VP16 in the prey context with LexA-Bas1 as bait; nonetheless, we could not detect an interaction with LexA-Bas1 (Fig. S3C). As noted by Stynen and colleagues, some expected interactions might not work in the Candida two-hybrid system due to intrinsic limitations of fusion proteins (18). Overall, we were not able to use the Candida two-hybrid approach to map the Grf10 interaction domain.

10.1128/mSphere.00467-18.6 FIG S3 The *Candida* two-hybrid assay did not detect interaction between Grf10 and Bas1. (A) Two-hybrid positive and negative controls, from Stynen et al. (18). LexA or LexA-Cph1 with Cek2-VP16 or VP16. (B) Strains expressing the bait constructs LexA, LexA-IR5, LexA-IRC100, and LexA-NIRC were transformed with prey constructs VP16 (empty vector) or Bas1-VP16, as indicated; all strains were derived from SC2H3. Serial dilutions (1:10) were plated on the indicated media (SC, SC-His-Met+Ade, and SC-His-Met-Ade), performed as described in Fig. 2. Plates were incubated at 30°C and photographed at 48 h. (C) Strains expressing baits LexA or LexA fused with full-length Bas1 (LexA-Bas1) were transformed with prey constructs VP16 or VP16 fused with full-length Grf10 (Grf10-VP16). Strains were prepared, spotted onto SC and SC-His-Met-Ade, and photographed as described above. At least three replicates were performed for each experiment. Download FIG S3, PDF file, 0.6 MB. Copyright © 2018 Wangsanut et al. 2018 Wangsanut et al. This content is distributed under the terms of the Creative Commons Attribution 4.0 International license.

### LexA-Grf10 displays adenine-responsive activation.

**FIG 3:**
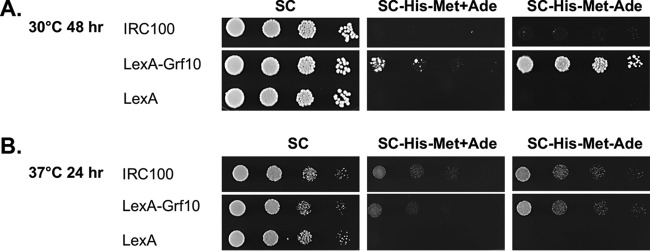
LexA-Grf10 displays adenine-responsive transactivation. Strains expressing LexA, LexA-Grf10, or IRC100 were grown as described in Fig. 2. Ten-fold serial dilutions were spotted onto SC and SC-His-Met with and without adenine, as indicated. (A) The plates were incubated at 30°C and photographed at 48 h. (B) The plates were incubated at 37°C and photographed at 24 h.

Grf10 is required for full expression of *ADE* and one-carbon metabolism genes in the absence of adenine, but it does not affect basal expression in the presence of adenine (12). Thus, we hypothesized that the ability of LexA-Grf10 to activate transcription is dependent on adenine limitation. To test this, we examined the ability of the LexA-Grf10 fragments and full-length fusion proteins to express *lexA_op_-HIS1* in the presence and absence of adenine. The carboxy-terminal constructs, Cterm1, Cterm2, and Cterm3, did not show any differences in their ability to activate transcription in the presence of adenine (Fig. 2C) from that in its absence (data not shown). The full-length LexA-Grf10 fusion protein was responsive to adenine, more strongly activating transcription of the *lexA_op_-HIS1* reporter at 30°C when adenine was limited (Fig. 3A); both the full-length LexA-Grf10 and IRC100 showed adenine-responsive activation at 37°C (Fig. 3B). The results indicate that Grf10 functions as a stronger activator when adenine is depleted and suggest that Grf10 activation activity is normally masked, likely by the IR, under adenine-repressing conditions.

### Adenine-responsive transactivation by LexA-Grf10 is independent of Bas1.

**FIG 4:**
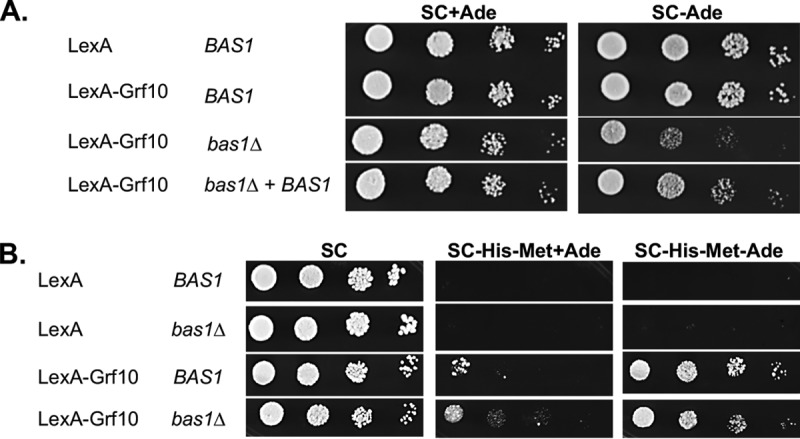
Adenine-dependent activation by LexA-Grf10 does not require Bas1. (A) Strains RAC201, RAC216, RAC290, and RAC291, with their relevant genotypes indicated, were assessed for adenine prototrophy on SC medium containing or lack adenine, as indicated. Plates were incubated at 30°C for 33 h. (B) Strains RAC201, RAC287, RAC216, and RAC290 were grown and diluted as described in Fig. 2 and plated on the media as indicated. These experiments were performed with at least three independent transformants.

We hypothesized that Bas1 is required for the adenine-responsive activation by LexA-Grf10, given that both Grf10 and Bas1 are necessary for *ADE* gene transcription (12) and the absence of adenine-regulated *Sc*Bas1-*Sc*Pho2 interaction in S. cerevisiae (32, 33). To do this, we deleted both alleles of *BAS1* in the *lexA_op_-HIS1* reporter strain and confirmed that the *bas1*Δ mutant exhibited adenine auxotrophy that was reversed by the restoration of *BAS1* or adenine supplementation (Fig. 4A).

Unexpectedly, LexA-Grf10 responded to adenine levels to activate the *lexA_op_-HIS1* reporter whether or not Bas1 was present (Fig. 4B). There was an increase in the basal expression of *HIS1* that was associated with *ARG4* (Fig. S4), suggesting increased basal promoter activity (from *ADH1*) or weakened adenine repression in the LexA fusion protein. Although basal expression of the *lexA_op_-HIS1* reporter increased, the adenine-dependent regulated expression of this reporter by LexA-Grf10 is independent of Bas1. This result indicates that the adenine signal is transmitted directly to Grf10 without requiring Bas1.

10.1128/mSphere.00467-18.7 FIG S4 *ARG4* restoration affects basal *lexAop-HIS1* reporter expression but not adenine-dependent activation by LexA-Grf10. C. albicans strains were spotted on the indicated media and incubated at 30°C, as described in Fig. 2. *BAS1* strains expressing LexA (RAC201), LexA-Grf10 (RAC216), or LexA-Grf10 with VP16 (RAC218), *BAS1/bas1Δ::ARG4* expressing LexA (RAC292) or LexA-Grf10 (RAC293), and *BAS1*-restored strain expressing LexA-Grf10 (RAC295) are shown; at least 3 independent isolates of each strain were examined. Plates were photographed at 48 h. Note that the pictures for the *BAS1* strain RAC216 are the same pictures as shown in Fig. 4B. Download FIG S4, PDF file, 0.5 MB. Copyright © 2018 Wangsanut et al. 2018 Wangsanut et al. This content is distributed under the terms of the Creative Commons Attribution 4.0 International license.

### Overexpression of LexA-Grf10 promotes filamentation and is dependent on the homeodomain.

**FIG 5:**
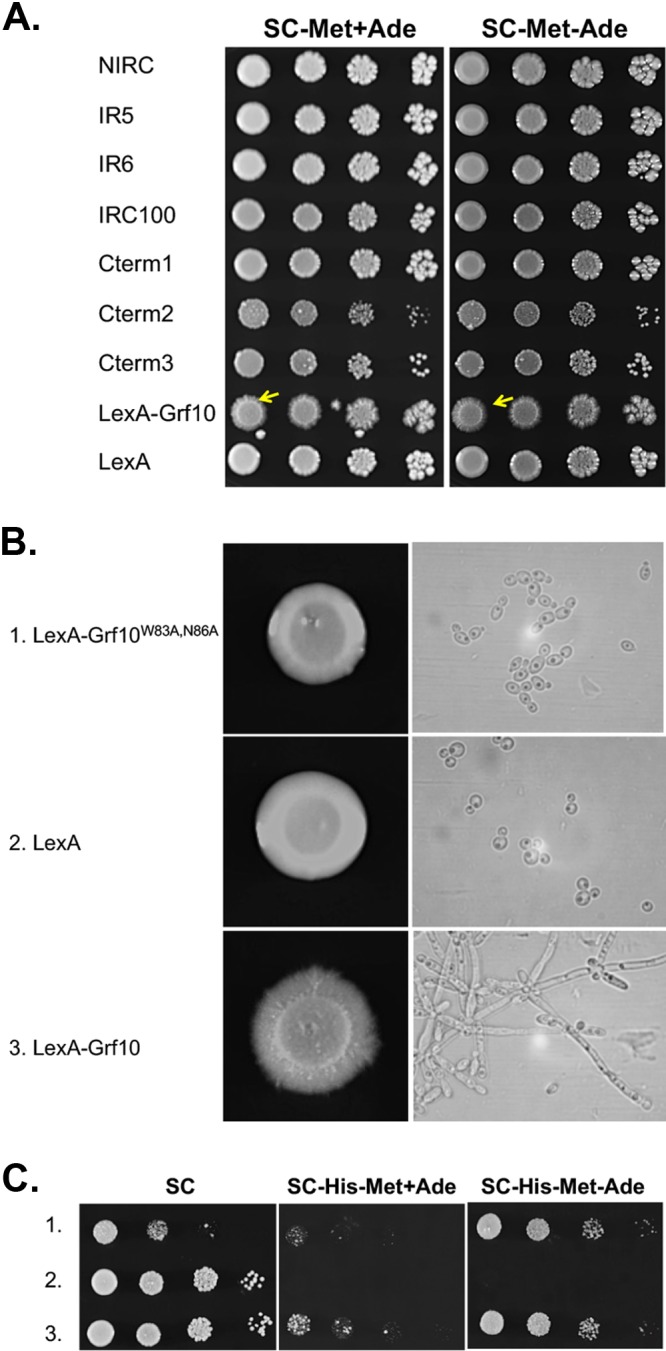
LexA-Grf10 overexpression triggers filamentation and requires a functional DNA-binding homeodomain. (A) Strains harboring the truncation and full-length LexA-Grf10 fusion constructs, as in Fig. 2, were grown on SC-Met or SC-Met−Ade medium for overexpression, as indicated. Yellow arrowheads indicate hyphal growth. Plates were incubated at 30°C, and photographs were taken at 72 h. (B) Left, strains expressing LexA-Grf10^W83A-N86A^ (1, RAC300), LexA (2, RAC201), or LexA-Grf10 (3, RAC216) were grown overnight in YPD and normalized to an OD_600_ of 0.1, and 3 µl were spotted onto solid SC-Met medium. Plates were incubated at 30°C, and pictures were taken at 96 h. Right, a single colony of each strain was inoculated into SC-Met medium and incubated for 16 h at 30°C. Samples were examined by light microscopy at ×100 magnification. (C) Strains numbered as in panel B were spotted on solid medium, as indicated and as described in Fig. 2. Plates were incubated at 30°C, and pictures were taken at 48 h. At least three biological replicates were performed for each experiment.

We observed that colonies displayed rough edges when the LexA-Grf10 full-length protein was overexpressed (Fig. 5A), suggesting an induction of filamentation under yeast growth conditions. We found increased hyphal formation on solid agar plates and in liquid broth when LexA-Grf10 was overexpressed (Fig. 5A and B). None of the one-hybrid LexA-fusion constructs were able to induce filamentation (Fig. 5A). Thus, these results indicated that overexpression of the entire Grf10 protein is required to drive filamentation and suggested that the LexA-Grf10 protein is going to other gene targets (outside *lexA_op_-HIS1*) to promote filamentation.

We hypothesized that this filamentation is dependent on the homeodomain of Grf10 within the LexA-Grf10 fusion protein. To test this, we mutated two conserved residues within the homeodomain to alanine; these amino acids, W83 and N86, recognize DNA and have been shown in *Sc*Pho2 to be critical for DNA binding and subsequent transcriptional activation (32, 34). The alanine substitutions did not change the protein stability (data not shown). LexA-Grf10 and LexA-Grf10^W83A-N86A^ promoted adenine-regulated expression of *lexA_op_-HIS1* to the same extent, indicating that the homeodomain is not required at this locus (Fig. 5C). Importantly, overexpression of the *lexA-grf10^W83A-N86A^* mutant completely failed to promote filamentation both on SC-Met solid and liquid media (Fig. 5B). These results showed that overexpression of LexA-Grf10 affects expression from other native promoters beyond *lexA_op_-HIS1* due to its own DNA-binding homeodomain.

### Filamentation driven by LexA-Grf10 overexpression is independent of Bas1 and adenine.

**FIG 6:**
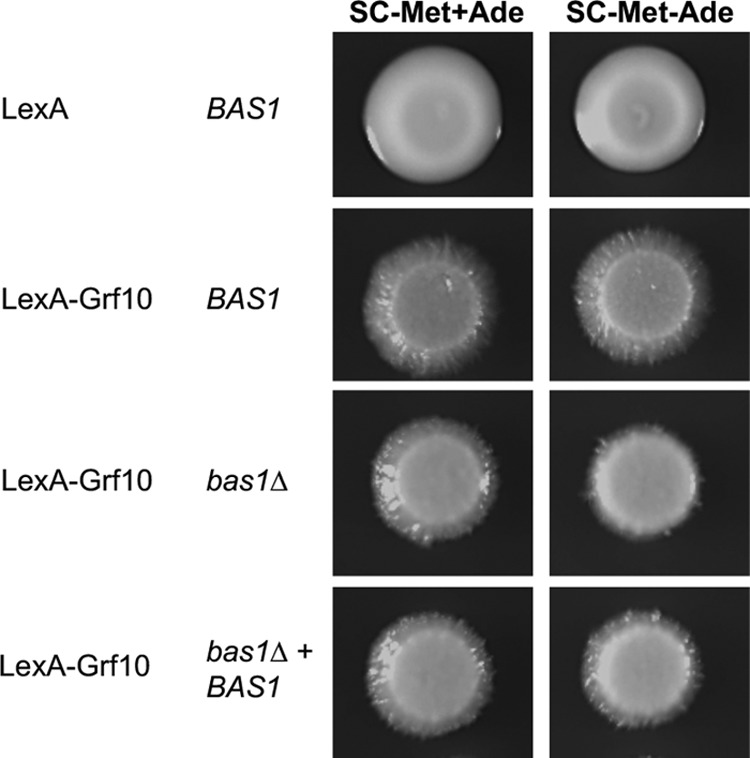
Filamentation produced by LexA-Grf10 overexpression is independent of Bas1 and adenine levels. *BAS1* (RAC201 and RAC216), *bas1*Δ (RAC290), and *BAS1*-restored (RAC291) strains harboring LexA and LexA-Grf10 were prepared as in Fig. 2 and spotted onto the indicated media. Plates were incubated at 30°C, and pictures were taken at 96 h. At least three biological replicates were performed per each experiment.

We wondered whether Bas1 was required for the induction of filamentation by LexA-Grf10. To test this, we overexpressed LexA-Grf10 in the *BAS1*, *bas1*Δ, and *BAS1-*restored mutant strains and assessed filamentation. LexA-Grf10 overexpression triggered filamentation independent of adenine (Fig. 6). In the *bas1*Δ mutant strain, LexA-Grf10 overexpression induced filamentation when adenine was provided (Fig. 6). When both adenine and Bas1 were absent, there were fewer hyphae produced, and this filamentation defect was reversed by the restoration of *BAS1*. This defect is likely a hyphal slow-growth phenotype, consistent with the slow growth of the yeast form *bas1*Δ mutant under adenine limitation (12). These data indicate that filamentation due to the overexpression of LexA-Grf10 is independent of Bas1, provided that there is sufficient adenine in the medium to support cell growth.

### Residues D302 and E305 within the IR are important for activation by Grf10.

Overexpression of LexA-Grf10 induces filamentation, and this requires its ability to bind DNA through the homeodomain; interaction with partner proteins is necessary for high-affinity DNA binding by *Sc*Pho2 (21, 22, 33, 35). Therefore, we hypothesize that the IR of Grf10 is important for the same function. Key amino acids within this region are predicted to be critical for partner interaction. To test this, we generated substitution mutations in three conserved amino acids within the IR core (see Fig. 1B), changing D302, E305, and Q308 to alanine. The mutations were examined (i) as LexA-Grf10 fusion proteins and (ii) as Grf10 isoforms integrated into the *grf10*Δ null mutant strain at the native locus and expressed from the native promoter.

**FIG 7:**
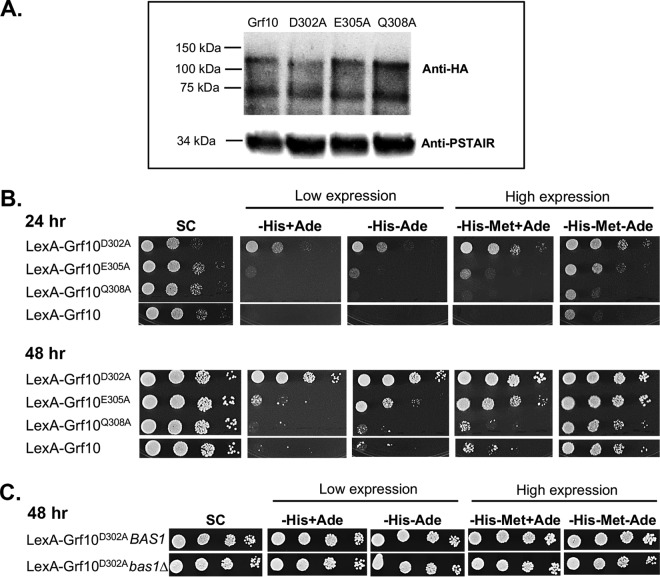
Grf10 IR residues are important for adenine limitation sensing and activation by LexA-Grf10. (A) Immunoblot to detect stability of the alanine substitution mutations in LexA-Grf10. Cells were grown in SC-Met medium. Immunoblotting was performed as described in Fig. 2. Top, immunoblot assay using anti-HA monoclonal antibody; bottom, anti-PSTAIR antibody as protein loading control. (B) Strains expressing LexA-Grf10 or mutated proteins LexA-Grf10^D302A^, LexA-Grf10^E305A^, and LexA-Grf10^Q308A^ were grown, diluted, and plated on the indicated medium, as described in Fig. 2. Top set shows growth after 24 h, and the bottom set shows growth after 48 h at 30°C. (C) Strains expressing LexA-Grf10^D302A^ in the *BAS1* or *bas1*Δ background were plated on SC medium, as indicated. Plates were incubated at 30°C and photographed at 48 h. Three biological replicates were performed per each experiment in panels B and C.

In the LexA fusion context, the alanine substitutions did not alter the protein levels (Fig. 7A), indicating that these mutations do not affect protein stability. The LexA-Grf10^D302A^ and LexA-Grf10^E305A^ proteins promoted transcription of the *lexA_op_-HIS1* reporter at higher levels than LexA-Grf10, whereas the LexA-Grf10^Q308A^ protein transcription was no different from that of LexA-Grf10 (Fig. 7B). Interestingly, transcription of *HIS1* by LexA-Grf10^D302A^ was not adenine repressible at either moderate or induced levels of expression, but transcription dependent on the LexA-Grf10^E305A^ protein was still adenine repressible (see high expression at 24 h and low expression at 48 h). The deletion of *BAS1* did not affect the high activation of the *lexA_op_-HIS1* reporter by LexA-Grf10^D302A^ (Fig. 7C), supporting a critical role for D302 in generating an adenine response in Grf10.

**FIG 8:**
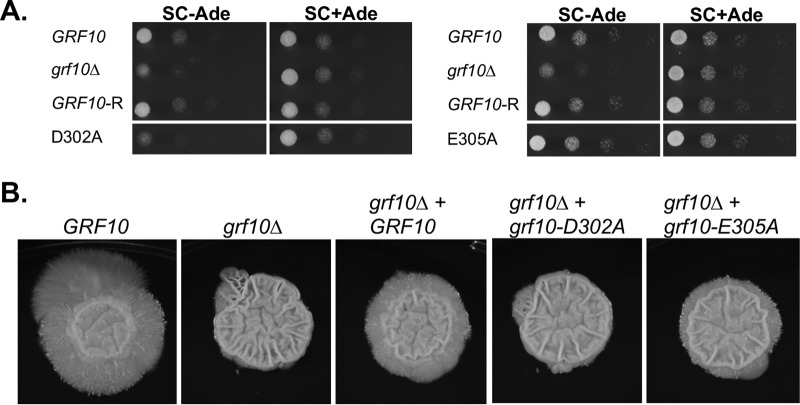
IR residues are necessary for native Grf10 activity during the response to adenine limitation and filamentation. (A) The diploid *GRF10* strain (DAY286), the *grf10*Δ null strain (RAC117), the heterozygous *GRF10* restored strain (*GRF10*-R; RAC120), and heterozygous restored strains carrying either the *grf10-D302A* allele (left, RAC259) or the *grf10-E305A* allele (right, RAC260) were grown overnight in YPD and the serially diluted cultures as described in Fig. 2. Cells were spotted on SC with or without adenine, and the plates were incubated at 30°C and pictured at 16 h. (B) The same strains were grown overnight in YPD and spotted on solid spider medium. The plates were incubated at 37°C and photographed weekly for up to 14 days. Representative samples are shown, repeated in duplicate. Measurements of the central and peripheral regions were performed as previously described (Ghosh et al. [11]).

In the native protein context, we examined the ability of the Grf10 mutants to promote growth on medium lacking adenine. The *grf10-*D302A allele failed to complement the growth defect of the *grf10*Δ mutant when adenine was limited (Fig. 8A, left). This result was intriguing because of the results described above that showed constitutive activation of *lexA_op_-HIS1* by LexA-Grf10^D302A^; this suggests that D302 is involved in additional activities beyond masking the activation domain. Conversely, the *grf10-*E305A allele was able to restore the prototrophy of the *grf10*Δ mutant (Fig. 8A, right).

Second, we assessed the ability of the *grf10-*D302A and *grf10-*E305A alleles to restore the filamentation defect found in the *grf10*Δ mutant. On solid spider medium, the *grf10*Δ mutant forms a wrinkled central region but lacks the peripheral filamentous region seen in the wild type, and the restored heterozygous strain exhibited shortened peripheral hyphae (Fig. 8B), as previously reported (11). Under these conditions, the peripheral hyphae of the restored strain contribute to ∼39% of the macrocolony’s diameter. We found that the *grf10-*D302A restored strain lacked the peripheral hyphae (Fig. 8B), indicating that this allele behaved as a null allele with regard to filamentation. The *grf10-*E305A allele formed a macrocolony with dramatically shortened peripheral hyphae that accounted for ∼20% of the diameter; thus, this allele partially restored the hyphal defect of the *grf10*Δ mutant (Fig. 8B). Together, these results showed different specific phenotypic effects of substitutions in the IR and demonstrated a critical role for D302 in filamentation and *ADE* gene regulation.

## DISCUSSION

Grf10 is a homeodomain transcription factor unique among other fungal homeodomain-containing transcription factors, such as Ste12 and Mata1/Matα2 (reviewed in references 36–39), because it regulates both morphology (yeast versus hyphae) and metabolic pathways. In this study, we characterized the functional domains of Grf10 and identified key residues that contribute to responses to adenine limitation and filamentation cues. We mapped the Grf10 activation domains to the C-terminal half of the protein, with two smaller regions, AD1 and AD2, that each contain multiple activation domain motifs, one of which, AD1, showed temperature-dependent activation at 37°C. This temperature effect may be due to altered conformational changes in Grf10 or in another protein that affects the ability of Grf10 to transactivate. High temperature (37°C) is well known to be a critical factor for the dimorphic transition in C. albicans (reviewed in reference 40); however, the basis of this temperature effect on filamentation remains enigmatic. Direct effects of temperature on transcription factors could be a cellular mechanism that promotes the hyphal transition.

Our data are consistent with a model in which LexA-Grf10 is folded into an inactive transcription factor, bound to DNA through the strong LexA DNA-binding domain; the CR region (residues 152 to 270) inhibits transactivation by AD1 in the context of the LexA-NIRC protein and might do so in the full-length protein. The IR also mediates inhibition; D302 is critical for masking the activation domains and E305 may play a similar but smaller role. In the native protein, the roles for these amino acids of the IR are more pivotal and complex. The Grf10^D302A^ protein, and to a lesser extent the Grf10^E305A^ protein, failed to respond to the adenine limitation and hypha-inducing cues, consistent with the roles for these amino acids in IR to interact with coregulators (Bas1 for adenine and unknown protein[s] during filamentation) in order to form a stable ternary complex with DNA, similar to what has been seen in S. cerevisiae (22, 33, 35, 41).

We also found differences in the response to adenine levels between Grf10 and *Sc*Pho2 as revealed by LexA fusion protein assays. In S. cerevisiae, LexA-Pho2 confers constitutively high levels of transcription, independent of adenine levels, as measured by β-galactosidase reporter assays (32). However, in this study, LexA-Grf10 in C. albicans displayed adenine-responsive activation of the *HIS1* reporter, leading to growth differences. Neither fusion protein required their Bas1 partner protein for expression of the reporter; the lack of dependence on the Bas1 proteins is likely due to the strength of the LexA DNA-binding domain-*lexA_op_* interaction. The lack of growth on SC-His+Ade medium versus the high expression of the *lacZ* reporter, in the face of constitutive DNA binding, indicates that masking of the activation domains in LexA-Grf10 occurs to a greater extent than in LexA-Pho2.

The alanine substitutions, homeodomain mutation, and *BAS1* deletion analyses revealed that Grf10 controls the filamentation and adenine limitation responses separately. Overexpression of LexA-Grf10 (Fig. 5 and 6) or of Grf10 (16) promotes hyphal formation; because the *GRF10* gene is normally upregulated under hypha-inducing conditions and during biofilm formation (11, 15), it is reasonable to think that overexpression of *GRF10* mimics this upregulation to further hyphal formation. In S. cerevisiae, *Sc*Bas1 and *Sc*Pho4 compete to interact with Pho2, leading to coordinated regulation of *de novo* purine biosynthesis with phosphate homeostasis (32, 41). By analogy, it is possible that Bas1 and other unknown protein partner(s) compete for Grf10; increased expression of Grf10 during filamentation could alter the competition dynamics, allowing for coordinated expression of hyphal growth and cellular nucleotide synthesis in C. albicans. Future studies will shed light on novel *GRF10* target genes and additional Grf10 protein partners that regulate filamentation and reveal if competition for Grf10 by protein partners leads to coordinated regulation of filamentation and purine metabolism in C. albicans.

Interestingly, Grahl and colleagues demonstrated that the level of signaling through the Ras/protein kinase A (Ras/PKA) pathway depends on intracellular ATP levels, such that low levels of ATP override hypha-inducing cues to prevent filamentation (42). ATP levels also modulate flux through the *de novo* purine biosynthesis pathway by feedback inhibition in S. cerevisiae (43). Low ATP levels generate a biosynthetic intermediate, AICAR (5-amino-4-imidazole carboxamide ribotide), which signals adenylate limitation to increase *ADE* gene expression (41, 43). AICAR stimulates an interaction between *Sc*Bas1 and *Sc*Pho2 (41), stabilizes *Sc*Pho2 binding to DNA (35), and results in increased gene expression (35, 43). Grf10 is responsive to the adenine limitation signal and thus may coregulate filamentation with intracellular adenylate pools to ensure sufficient nucleotides and cellular energy for hyphal growth.

We found that there are several ways for C. albicans to regulate Grf10, including masking Grf10 activation domains, altering the ability to transactivate by temperature, increasing the expression of *GRF10*, and changing interacting protein partners. The multiple layers of Grf10 activity regulation underscore the importance of Grf10 in the precise regulation of C. albicans cellular processes. Grf10 has orthologues in many fungal species, including other plant and animal pathogens, such as Aspergillus (both A. nidulans and A. fumigatus), rice blast fungus (Magnaporthe oryzae), gray mold disease (Botrytis cinerea), and corn smut (Ustilago maydis). We suggest that the conserved IR in these orthologues is important for protein partner interactions that could regulate morphogenesis and metabolism. Thus, this study provides fundamental knowledge that could potentially lead to the development of novel therapeutic interventions not only for C. albicans, but also for other fungal pathogens.

## MATERIALS AND METHODS

### Strains and growth media.

Candida albicans strains were grown in YPD (1% yeast extract, 2% peptone, and 2% dextrose) or SC medium (2% dextrose, 0.5% ammonium sulfate, 0.17% yeast nitrogen base, supplemented with one of the amino acid mixes CSM-Met, CSM-His-Ade-Met, CSM-Leu, CSM-His, CSM-Ade or CSM-Arg [Sunrise Science Products or MP Biologicals]) (44); histidine (0.3 mM), methionine (0.3 mM), and adenine (0.3 mM) were added as indicated in the figures. Hyphal formation was monitored on solid spider medium (45). Strains were maintained at room temperature on YPD plates and restreaked weekly from frozen stocks.

The strains of C. albicans used and generated in this study are listed in Table S1 (46–48). Derivatives of strain SC2H3 that express a LexA-fusion bait plasmid (Table S2, construction described below) were generated by linearizing each plasmid with NotI and transforming it into strain SC2H3; selection was made on SC-Leu medium (18). Correct plasmid integration between the *XOG1* and *HOL1* genes in selected transformants was verified by diagnostic PCR using bait integration primers (Table S3) that yielded a 2.2-kb product.

10.1128/mSphere.00467-18.1 TABLE S1 Strains. Download Table S1, DOCX file, 0.02 MB. Copyright © 2018 Wangsanut et al. 2018 Wangsanut et al. This content is distributed under the terms of the Creative Commons Attribution 4.0 International license.

10.1128/mSphere.00467-18.2 TABLE S2 Plasmids. Download Table S2, DOCX file, 0.02 MB. Copyright © 2018 Wangsanut et al. 2018 Wangsanut et al. This content is distributed under the terms of the Creative Commons Attribution 4.0 International license.

10.1128/mSphere.00467-18.3 TABLE S3 Primers. Download Table S3, DOCX file, 0.01 MB. Copyright © 2018 Wangsanut et al. 2018 Wangsanut et al. This content is distributed under the terms of the Creative Commons Attribution 4.0 International license.

Strains RAC259 and RAC260 express the *grf10-*D302A and *grf10-*E305A mutant alleles, respectively, at the native locus, and were constructed by restoring the allele into the *grf10*Δ mutant strain RAC117, as described previously (11). Plasmids pGEM-D302A and pGEM-E305A were linearized with Bpu10I and used to transform RAC117, selecting for histidine prototrophy on SC-His medium. Correct plasmid integration was verified by diagnostic PCR using primers US600-GRF10-F and HIS-R.

*BAS1* was deleted from strain SC2H3 in two steps. The *bas1*Δ::*ARG4* allele was amplified from the genomic DNA of RAC108 using primers BAS1-DF and BAS1-DR (12); this DNA transformed SC2H3, selecting on SC-Arg medium. Confirmation of the *BAS1/bas1*Δ::*ARG4* heterozygous genotype in RAC285 was made by using primers BAS1-500-US-F and BAS1-500-DS-R. The *bas1*Δ::*SAT1* flipper allele was amplified from plasmid pSFS2A (Reuss et al. [49]) using primers BAS1-KO-F and BAS1-KO-R and used to transform strain RAC285, selecting for nourseothricin resistance. Confirmation of the *bas1*Δ::*ARG4*/*bas1*Δ::*SAT1* flipper genotype in strain RAC286 was performed using primers BAS1-500-US-F and BAS1-500-DS-R.

Strain RAC286 was transformed with plasmids pC2HB (empty bait), pC2HB-*GRF10*, and pC2HB-*grf10-D302A* (selection for leucine prototrophy and integration was confirmed as described below) to generate strains RAC287, RAC288, and RAC289, respectively. The Sat1-flipper cassette was excised from RAC288, as described previously (49), generating strain RAC290. *BAS1* was restored in strain RAC290 using PshAI-linearized pSFS2A-*BAS1*, as described previously (12), generating strain RAC291.

### Plasmids.

Plasmid pC2HB (18) was modified to contain various *GRF10* fragments or *GRF10* point mutated alleles. To generate constructs IR5, IR6, IRC100, NIRC, Cterm1, Cterm2, Cterm3, and full length, *GRF10* fragments were PCR amplified (PrimeStar from TaKaRa or OneTaq from NEB) using genomic DNA prepared from C. albicans strain SC5314 and primers listed in Table S3. The *grf10-*D302A, *grf10-*E305A, and *GRF10-*Q308A alleles were generated by fusion PCR (PrimeStar), using overlapping mutagenic primers (Table S3) and pC2HB-GRF10 as the DNA template. All PCR-amplified *GRF10* was ligated into pC2HB at the AscI and NheI restriction sites and transformed into E. coli DH5α competent cells. The LexA-GRF10 fusion plasmids were sequenced (Genewiz).

To generate plasmid pGEM-D302A and pGEM-E305A, the *grf10-*D302A and *grf10-*E305A alleles generated by fusion PCR from previous step were moved into plasmid pGHPF (12) using a gap-repair cloning approach (50). Briefly, plasmid pGHPF, which carries *GRF10* (11), was digested with BstBI and PshAI, and the large fragment was separated from the small 0.7-kb fragment by gel electrophoresis and was purified (Qiagen gel extraction kit). This gapped pGHPF plasmid was mixed with the mutated *grf10* PCR fragments and transformed into E. coli DH5α competent cells (50). Plasmids were isolated and their sequences determined (Genewiz) before transformation.

### Growth assays.

To assay growth dependent on the expression of *lexA_op_-HIS1,* strains were grown overnight in 5 ml of YPD broth and normalized to an optical density at 600 nm (OD_600_) of 0.1 in sterile water. Ten-fold serial dilutions were made in sterile water, and 3 µl was spotted on SC medium supplemented as indicated in the figures. The plates were incubated at 30°C or 37°C, as noted in the figures. To assay hyphal induction, strains were spotted onto spider medium, as previously described (12).

### Protein extraction and Western blot analysis.

Strains were grown overnight in YPD at 30°C. The overnight culture was then inoculated 1:50 into fresh YPD medium and grown to mid-log phase (OD_600_, 0.5 to 1). The log-phase culture was normalized to OD_600_ of 1, and 5 ml of this culture was pelleted by centrifugation at low speed (770 × *g*, 2 min) and washed twice with SC-Met (for overexpression) or SC (for moderate expression). This normalized culture was then suspended in the corresponding SC or SC-Met medium and incubated for 30 min at 30°C in a shaking incubator. After 30 min, the cultures were quickly chilled in an ice-water bath, pelleted by centrifugation at 4°C and 770 × *g* for 2 min, and stored at -80°C.

The protein extraction was performed as described previously (16, 51). Briefly, the frozen cell pellet was resuspended in 200 µl of lysis buffer (0.1 M NaOH, 0.5 M EDTA, 2% SDS, 2% β-mercaptoethanol, protease inhibitor cocktail mix [Thermo Scientific]), and incubated for 10 min at 90°C. Five microliters of 4 M acetic acid was added to the cell lysate to neutralize the pH. After 10 min of incubation at 90°C, 50 µl of loading buffer (0.25 M Tris-HCl [pH 6.8], 50% glycerol, 0.05% bromophenol blue) was added and centrifuged at 16,000 × *g* for 2 min to obtain a clarified lysate. Proteins in the lysate were separated on Mini-Protean precast TGX gel (Bio-Rad) and transferred onto nitrocellulose membranes. The hemagglutinin (HA) epitope tag was detected using monoclonal anti-HA (BioLegend); anti-PSTAIR (Sigma) was used as protein loading control. After primary antibody incubation, the horseradish peroxidase-conjugated goat anti-mouse antibody (Bio-Rad) was added to the membrane, and the ECL detection kit (GE Amersham) was used for protein detection. Immunoblots were imaged using the chemiluminescent option in ImageQuant LAS 4000 imager.

### *In silico* protein prediction assessments.

Grf10 and *Sc*Pho2 protein sequences, retrieved from the Candida Genome Database and Saccharomyces Genome Database (19, 52, 53), respectively, were aligned using the SIM Alignment tool (https://web.expasy.org/sim/) (54), and a graphical representation of Grf10 and *Sc*Pho2 alignment was generated using the LALNVIEW program (55). To identify homologues of *GRF10* in other species or orthologues that carried the IR, the entire protein sequences corresponding to Grf10 or only the IR amino acid sequences from *Sc*Pho2, as defined by Bhoite et al. (22), were compared with the nonredundant protein sequences of selected fungal species, using the BLAST search tool (19, 52, 56). The selected ascomycete species chosen included only those species in which there are published reports on the putative ortholog (Table 1); however, sequences corresponding to the outlier basidiomycetes are uncharacterized. Protein sequence alignment of Grf10 homologous proteins was analyzed by using ClustalX version 2.1 (57). The phylogenetic tree was developed by using the bootstrap neighbor-joining (N-J) tree function in ClustalX 2.1, with 1,000 bootstraps, and viewed in MEGA7 (58) using Root on midpoint parameter.

To analyze Grf10 activation domains, the Grf10 entire protein sequence or portions of the sequence corresponding to the truncation fragments were characterized using the Motif Scan tool under the MyHits website, the PROSITE profiles as the motif source, and the 9aaTAD prediction tools using the moderate stringency criteria (27, 59). Website links are found in the legend for Fig. S2.
