# Erratum for Wangsanut et al., “Functional Mapping of Transcription Factor Grf10 That Regulates Adenine-Responsive and Filamentation Genes in *Candida albicans*”

**DOI:** 10.1128/mSphere.00094-19

**Published:** 2019-02-20

**Authors:** Tanaporn Wangsanut, Joshua M. Tobin, Ronda J. Rolfes

**Affiliations:** a Department of Biology, Georgetown University, Washington, DC, USA

## ERRATUM

Volume 3, no. 5, e00467-18, 2018, https://doi.org/10.1128/mSphere.00467-18. Supplemental Figure S3 legend: the description for panel B of this figure is incomplete. The last sentence of the description for this panel should read, “Plates were incubated at 30°C and photographed at 48 h; photos of LexA-IR5 with VP16 or Bas1-VP16 are from day 7.”

