# Supplementary figures and images for: Functional Mapping of Transcription Factor Grf10 That Regulates Adenine-Responsive and Filamentation Genes in Candida albicans
Source: mSphere. 2018 Oct 24;3(5):e00467-18. doi: 10.1128/mSphere.00467-18 (PMC6200990; doi:10.1128/mSphere.00467-18)

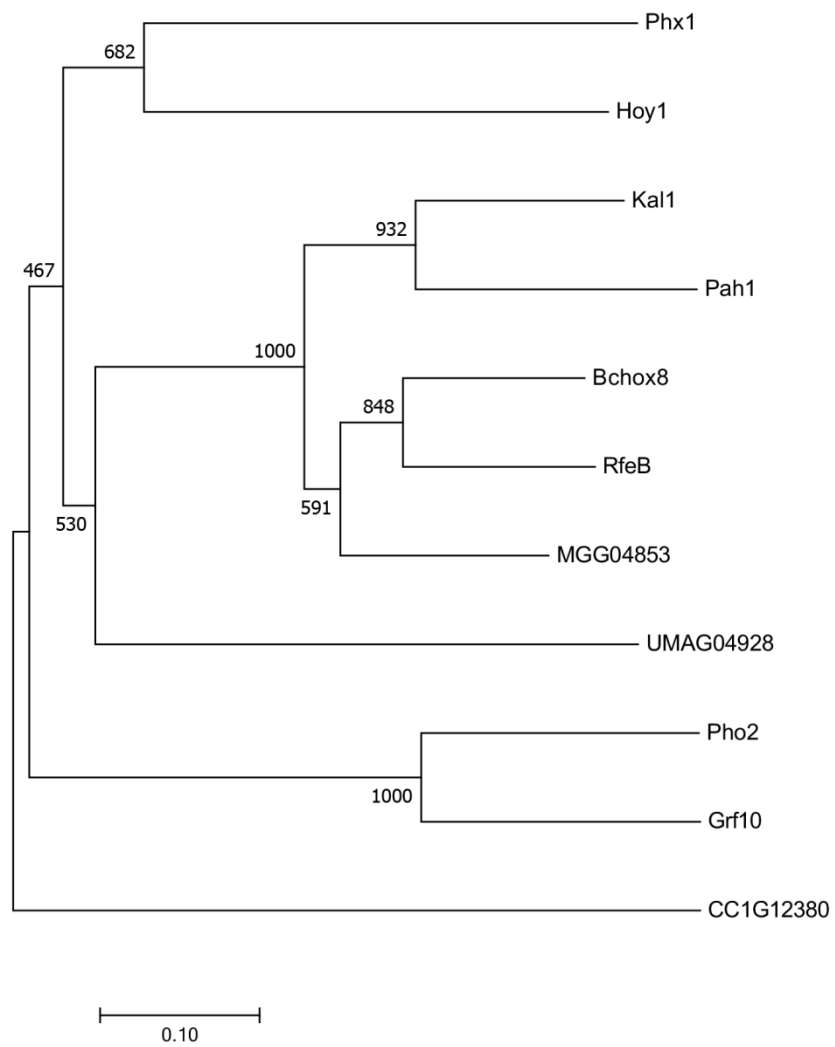

Supplement: FIG S1 [file sph005182666sf1.pdf]

**A.**

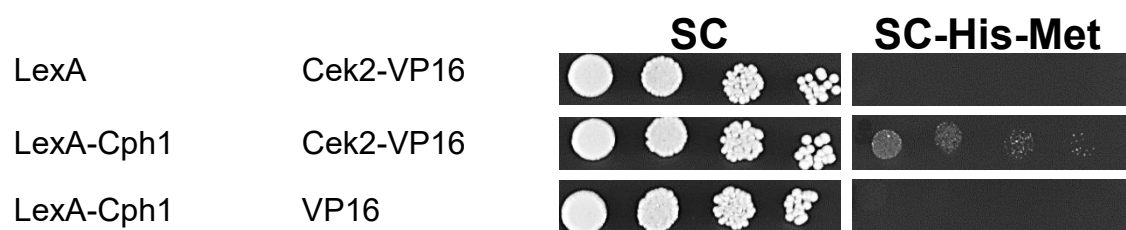

**B.**

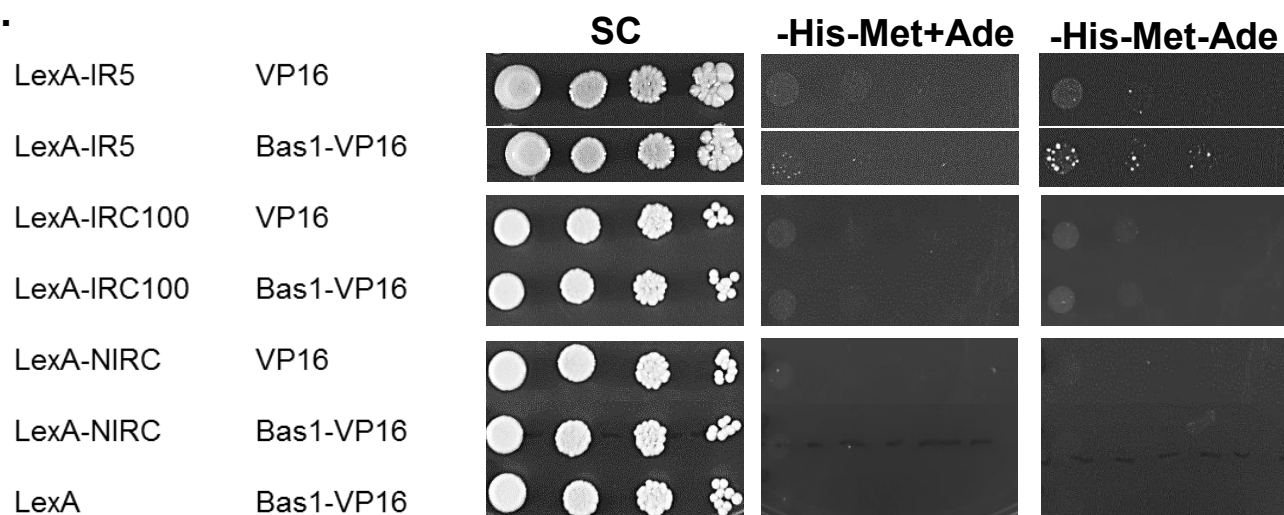

**C.**

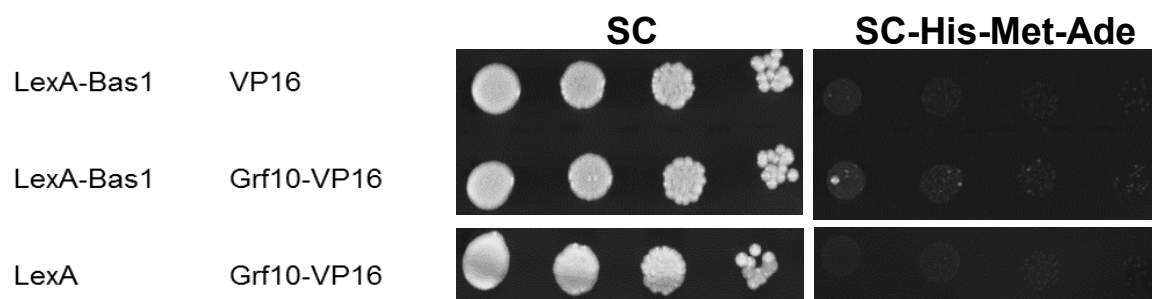

Supplement: FIG S3 [file sph005182666sf3.pdf]
