# Supplementary material for: Functional Mapping of Transcription Factor Grf10 That Regulates Adenine-Responsive and Filamentation Genes in Candida albicans
Source: mSphere. 2018 Oct 24;3(5):e00467-18. doi: 10.1128/mSphere.00467-18 (PMC6200990; doi:10.1128/mSphere.00467-18)
Supplement: FIG S2 [file sph005182666sf2.pdf]

A.

MSPDSISSSVNQDLSTPTPPTSLSSTSTNSNTNASSGQKRIRATGEALEFLISEFETNPNPSPE
RRKFISDKAQMNEKAVRIWFQNRRAKQRKFERQMLRKETDSPGNYAGIYNTYTPNPPTVTTDMT
NFNVNGSAGGATADFNDKLKNISSIPVEVNEKYCFIDCRSLSVGSWQRIKTGFHQSNLLTNNLI
NLAPVTLNQVMSNADLLIILSKKNLELNYFFSAISNNSRILFRIFYPLNAVVKCSLFDNNYYQN
NNTNGTNSSDDNNISEIRLNLCQKPKFSVYFFNGSNTNNQWSICDDFSEGQQVSCAFAANDNSL
PSNGKNQSFS**NTIPHVLVGSTLSLQYLSQFILQHQQRQRQQQRQAQPQPQPQPHNQFDFNSQPF** AD1
ETIPTSAINNNFNTTKTVNSSSMQGFVPGDFQDLPAIYESISNSNHTTTTDLKDTNATTTTTNK
HTPSSTTFTPQNLNGSNQSQTNVTYTNNYNESPFSIASTTNNNNTNSNRSNSQSHNPIFSDQLF
YESSESASTNSPQFAMKKMNSETKLYINGNVSSNSSTNGPPLDDDNN**LFDGVTRFTTTETSPED** AD2
DIIGMFTSQAHEPSAFELANGVTSSGSSYTHINNGSLTKSDKTFTGLSETSNNNNNTNGINFVD
DFHVGNEFGDIDFEHHYSQDHHQQQQKNDNNNGNTNLDSFIDFEN

B.

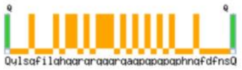

pos.: **345-382**
raw-score = **74**
N-score = **11.065**
E-value = **0.00018**

Glutamine-rich region profile.

C.

Sequence	Start	End	C1	C2	C3	C4	C5	C6	C7	C8	C9	C10	C11	C12	
DSISSSVNQ	4	12	-	-	+	-	+	+	-	+	+	-	+	+	58% match
SSVNQDLST	8	16	-	-	+	-	+	+	-	+	-	+	+	+	58% match
**EALEFLISE**	**46**	**54**	+	+	+	+	+	+	+	+	+	+	+	+	Perfect match
EFLISEFET	49	57	+	-	+	-	+	+	+	+	+	+	+	+	83% match
NLLTNNLIN	185	193	+	+	+	-	+	+	-	+	-	+	+	+	75% match
**NYFFSAISN**	**220**	**228**	+	+	+	+	+	+	+	+	+	+	+	+	Perfect match
QYLSQFILQ	345	353	+	+	+	+	+	+	+	+	-	+	+	+	92% match
SAINNNFNT	390	398	+	-	+	-	+	+	-	+	-	+	+	+	67% match
TNLDSFIDF	675	683	-	+	+	-	+	+	+	+	+	+	+	+	83% match
