# Supplementary material for: Functional Mapping of Transcription Factor Grf10 That Regulates Adenine-Responsive and Filamentation Genes in Candida albicans
Source: mSphere. 2018 Oct 24;3(5):e00467-18. doi: 10.1128/mSphere.00467-18 (PMC6200990; doi:10.1128/mSphere.00467-18)
Supplement: FIG S4 [file sph005182666sf4.pdf]

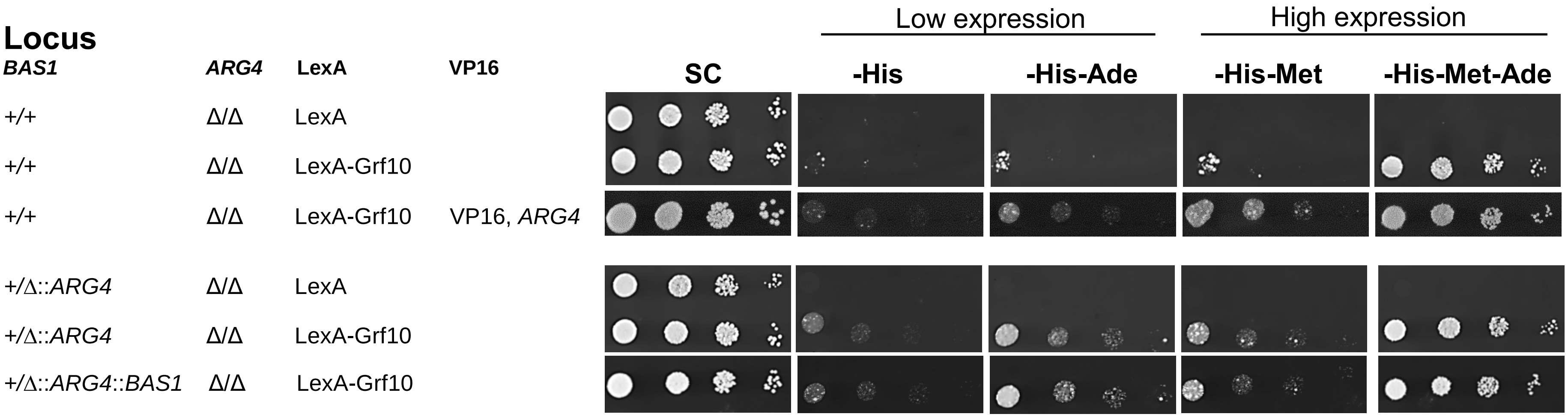

Locus
BAS1
ARG4
LexA
VP16
Low expression
High expression
SC
-His
-His-Ade
-His-Met
-His-Met-Ade
+/+ Δ/Δ LexA
+/+ Δ/Δ LexA-Grf10
+/+ Δ/Δ LexA-Grf10 VP16, ARG4
+/Δ::ARG4 Δ/Δ LexA
+/Δ::ARG4 Δ/Δ LexA-Grf10
+/Δ::ARG4::BAS1 Δ/Δ LexA-Grf10
