# Supplementary material for: Functional Mapping of Transcription Factor Grf10 That Regulates Adenine-Responsive and Filamentation Genes in Candida albicans
Source: mSphere. 2018 Oct 24;3(5):e00467-18. doi: 10.1128/mSphere.00467-18 (PMC6200990; doi:10.1128/mSphere.00467-18)
Supplement: TABLE S1 [file sph005182666st1.docx]

| **Name** | **Genotype** | **Source** |
| --- | --- | --- |
| SC5314 | *URA3/URA3* | Fonzi and Irwin, 1993 |
| SC2H3 | *arg4*Δ/*arg4*Δ *leu2*Δ/*leu2*Δ *his1*Δ*/his1*Δ *URA3/ura3*Δ *IRO1/iro1*Δ  *5xLexA_op_-ADH1b-HIS1 5xLexA_op_-ADH1b-lacZ* | Stynen et al., 2010 |
| RAC201 | SC2H3 ^a^ <*lexA*, *CmLEU2*> | This study |
| RAC214 | SC2H3 ^b^ <VP16, *CdARG4*> | This study |
| RAC230 | SC2H3 <*lexA*, *CmLEU2*> <*CEK2*-VP16, *CdARG4*> | This study |
| RAC231 | SC2H3 <*lexA-CPH1*, *CmLEU2*> <*CEK2*-VP16, *CdARG4*> | This study |
| RAC232 | SC2H3 <*lexA-CPH1*, *CmLEU2*> <VP16, *CdARG4*> | This study |
| RAC220 | SC2H3 <*lexA*-IRC100, *CmLEU2*> <VP16, *CdARG4*> | This study |
| RAC221 | SC2H3 <*lexA*-NIRC, *CmLEU2*> <VP16, *CdARG4*> | This study |
| RAC226 | SC2H3 <*lexA*-IR5, *CmLEU2*> <VP16, *CdARG4*> | This study |
| RAC227 | SC2H3 <*lexA*-IR6, *CmLEU2*> <VP16, *CdARG4*> | This study |
| RAC263 | SC2H3 <*lexA*-Cterm1, *CmLEU2*> | This study |
| RAC264 | SC2H3 <*lexA*-Cterm2, *CmLEU2*> | This study |
| RAC265 | SC2H3 <*lexA*-Cterm3, *CmLEU2*> | This study |
| RAC216 | SC2H3 <*lexA*-*GRF10*, *CmLEU2*> | This study |
| RAC218 | SC2H3<*lexA*-*GRF10*, *CmLEU2*> <VP16, *CdARG4*> | This study |
| RAC246 | SC2H3 <*lexA*-*grf10-D302A*, *CmLEU2*> | This study |
| RAC249 | SC2H3 <*lexA*-*grf10-E305A*, *CmLEU2*> | This study |
| RAC252 | SC2H3 <*lexA*-*GRF10-Q308A*, *CmLEU2*> | This study |
| RAC300 | SC2H3 <*lexA*-*grf10-W83A,N86A*, *CmLEU2*> | This study |
| RAC285 | SC2H3 *BAS1*/ *bas1∆*::*ARG4* | This study |
| RAC286 | SC2H3 *bas1∆*::*ARG4/bas1∆*::*SAT1-FLIP* | This study |
| RAC287 | SC2H3 *bas1∆*::*ARG4/bas1∆*::*SAT1-FLIP* <*lexA*, *CmLEU2*> | This study |
| RAC288 | SC2H3 *bas1∆*::*ARG4 bas1∆*::*SAT1-FLIP* <*lexA*-*GRF10*, *CmLEU2*> | This study |
| RAC289 | SC2H3 *bas1∆*::*ARG4 bas1∆*::*SAT1-FLIP* <*lexA*-*GRF10-D302A*, *CmLEU2*> | This study |
| RAC290 | SC2H3 *bas1∆*::*ARG4/bas1∆*::*FRT* <*lexA*-*GRF10*, *CmLEU2*> | This study |
| RAC291^c^ | SC2H3 *bas1∆*::*ARG4/bas1∆*::*FRT*::<*BAS1*, *SAT1-FLIP*> <*lexA*-*GRF10*, *CmLEU2*> | This study |
| RAC292 | SC2H3 *BAS1*/ *bas1∆*::*ARG4* <lexA, *CmLEU2*> | This study |
| RAC293 | SC2H3 *BAS1*/ *bas1∆*::*ARG4* <lexA-*GRF10*, *CmLEU2*> | This study |
| RAC295 | SC2H3 *BAS1*/ *bas1∆*::*ARG4*::<*BAS1*, *SAT1 FLIP*> <*lexA*-*GRF10*, *CmLEU2*> | This study |
| BWP17 | *ura3*::λ*imm434/ura3*::λ*imm434 his1*::*hisG/his1*::*hisG arg4*::*hisG/arg4*::*hisG* | Wilson et al. 1999 |
| DAY286 | BWP17 *ARG4::URA3::arg4::hisG/arg4::hisG* | Davis et al. 2000 |
| RAC117 | BWP17 *grf10∆::ARG4/grf10∆::URA3* | Ghosh et al. 2015 |
| RAC120 | BWP17 *grf10∆::ARG4/grf10∆::URA3::<GRF10, HIS1>* | Ghosh et al. 2015 |
| RAC259 | BWP17*grf10∆::ARG4/grf10∆::URA3::<grf10-D302A, HIS1>* | This study |
| RAC260 | BWP17 *grf10∆::ARG4/grf10∆::URA3::<grf10-E305A, HIS1>* | This study |

**Supplemental Table 1. *C. albicans* strains**

^a^ NotI site on chromosome 1 between *XOG1* and *HOL1* for integration of pC2HB and derivatives

^b^ NotI site on chromosome 2 between *RXT3* and orf19.3569 for integration of pC2HP and derivatives

^c^ The *bas1*Δ allele in which integration of <*BAS1, SAT1-FLIP>* occurred was not determined
