# Supplementary material for: Functional Mapping of Transcription Factor Grf10 That Regulates Adenine-Responsive and Filamentation Genes in Candida albicans
Source: mSphere. 2018 Oct 24;3(5):e00467-18. doi: 10.1128/mSphere.00467-18 (PMC6200990; doi:10.1128/mSphere.00467-18)
Supplement: TABLE S2 [file sph005182666st2.docx]

**Supplemental Table 2. Plasmids used**

| **Plasmid Name** | **Vector** | **Source** |
| --- | --- | --- |
| pC2HP | - | 18 |
| pC2HB | - | 18 |
| LexA-Cph1 | pC2HB | This study |
| LexA-Bas1 | pC2HB | This study |
| pC2HB-IR5 | pC2HB | This study |
| pC2HB-IR6 | pC2HB | This study |
| pC2HB-NIRC | pC2HB | This study |
| pC2HB-IRC100 | pC2HB | This study |
| pC2HB-Cterm1 | pC2HB | This study |
| pC2HB-Cterm2 | pC2HB | This study |
| pC2HB-Cterm3 | pC2HB | This study |
| pC2HB-GRF10FL | pC2HB | This study |
| pC2HB-GRF10^D302A^ | pC2HB | This study |
| pC2HB-GRF10^E305A^ | pC2HB | This study |
| pC2HB-GRF10^Q308A^ | pC2HB | This study |
| pC2HB-GRF10^W83A,N86A^ | pC2HB | This study |
| Cek2-VP16 | pC2HP | This study |
| Bas1-VP16 | pC2HP | This study |
| pGEM-HIS1 | - | 47 |
| pGHPF | pGEM-HIS1 | 12 |
| pGEM-D302A | pGHPF | This study |
| pGEM-E305A | pGHPF | This study |
| pSFS2A | - | 49 |
| pSFS2A-*BAS1* | - | 12 |
