# Supplementary material for: Functional Mapping of Transcription Factor Grf10 That Regulates Adenine-Responsive and Filamentation Genes in Candida albicans
Source: mSphere. 2018 Oct 24;3(5):e00467-18. doi: 10.1128/mSphere.00467-18 (PMC6200990; doi:10.1128/mSphere.00467-18)
Supplement: TABLE S3 [file sph005182666st3.docx]

**Supplemental Table 3. Oligonucleotides**

| **Primer** | **Description** | **Sequence 5'-3'** |
| --- | --- | --- |
| **LexA fusion** | | |
| 1-158F | Clone Grf10 into pC2HB | TAAGCAgctagcATGTCTCCTGACTCTATTTCATCACTG |
| GRF10FL-R |  | ACCACCggcgcgccTTAATTTTCAAAATCAATAAAACTGTCTAA |
| GRF10IR-F | Clone IRC100 into pC2HB | GCATgctagcTGTCAAAAACCCAAATTTCTGG |
| GRF10IR-C100-R |  | TGCTTAggcgcgccTTAATCAGTTGTGGTAGTGTGATTACTATT |
| N120-GRF10IR-F | Clone NIRC into pC2HB; with GRF10IR-C100R | TAAGCAgctagcGAGAAATATTGTTTTATTGATTGTCGTTCA |
| IR5-F | Clone IR5 into pC2HB | TAAGCAgctagcAGTATACCAGTTGAAGTGAATGAGAAATAT |
| IR5-R |  | TGCTTAggcgcgccTTATTGCAAAATGAATTGACTTAAATATTGTAA |
| IR6-F | Clone IR6 into pC2HB; with IR5 R | TAAGCAgctagcATTAGTGAAATTAGATTAAACTTGTGTCAA |
| 560-685-F | Clone 560-685 into pC2HB; with GRF10FL R | TAAGCAgctagcTTGTTTGACGGAGTCACAAGATTTACTACT |
| 436-560-AD-F | Clone 436-560 into pC2HB | TAAGCAgctagcTTAAAGGATACCAACGCCACCACC |
| 436-560-R |  | TGCTTAggcgcgccTTACAAATTATTATCGTCATCAAGAGGTGGGCC |
| 436-685-AD-R | Clone 436-685; with 436-560 F | ACCACCggcgcgccTTAATTTTCAAAATCAATAAAACTGTCTAAATT  AGTGTT |
| W83AN86A-F | Clone W83A,N86A into pC2HB | ATAAAGCTCAAATGAATGAGAAAGCCGTTCGAATTgcGTTCCAAgcTAGAAGAGCAAAAC |
| W83AN86A-R |  | CTGGCAAATCTTGGAAATCACCGGGAACAAATCCTTGCATGCTGGATGAATTGA |
| pC2HB seq F | pC2HB sequencing | tatccgtatgatgtgcctgac |
| pC2HB seq R |  | ccagatttccagatttccaga |
| Bait integration F | Check pC2HB integration | ATGAAAGGACAATCACGAAGCC |
| Bait integration R |  | CGGAGAACCTGCGTGCAATCC |
| ***GRF10* point mutated alleles** | | |
| D302A-F | Right fragment for fusion PCR; with GRF10FL R | ATCAATGGTCTATTTGTGAT**GCT**TTTAGTGAGGGTCAACAAGT |
| D302A-R | Left fragment for fusion PCR; with 1-158 F | ACTTGTTGACCCTCACTAAA**AGC**ATCACAAATAGACCATTGAT |
| E305A-F | Right fragment for fusion PCR; with GRF10FL R | CTATTTGTGATGATTTTAGT**GCG**GGTCAACAAGTGAGTTGTGC |
| E305A-R | Left fragment for fusion PCR; with 1-158 F | GCACAACTCACTTGTTGACC**CGC**ACTAAAATCATCACAAATAG |
| Q308A-F | Right fragment for fusion PCR; with GRF10FL R | ATGATTTTAGTGAGGGTCAA**GCA**GTGAGTTGTGCTTTTGCTGC |
| Q308A-R | Left fragment for fusion PCR; with 1-158 F | GCAGCAAAAGCACAACTCAC**TGC**TTGACCCTCACTAAAATCAT |
| US600-GRF10-F | Diagnostic PCR to check pGHPF integration | TTTGTTTACCTAGAATAACTTCAGTGTTGCTAGTGA |
| HIS1-R |  | GCTCGCGACACGTTTCAACGA |
| ***BAS1* Deletion** | | |
| BAS1-KO-F | Delete *BAS1* and mark with SAT1 flipper | TATCTATTCTGTGTATTTGTACACCTATAAATAAACAATATCAATATACTAGACAACAAG**CGGGCCCCCCCTCGAGGAAGT** |
| BAS1-KO-R |  | TTCTCATAAAACTTAAAATTATATTTCTATTAATCATTTCTACTAATTATAGATATACAT**CTCTAGAACTAGTGGATCTGA** |
| BAS1-DF | Amplify *bas1*∆::*ARG4* from RAC108 | GTGAAGTTTCTGATGCGAC |
| BAS1-DR |  | GCCAAGGGACCTATTTGC |
| BAS1-500-US-F | Diagnostic PCR to check *BAS1* deletion | AATTAAGAGCTGGTTTATATGTCC |
| BAS1-500-DS-R |  | ACGTTGTCGGAATTTATAAGTACC |
| BAS1P-F | Diagnostic PCR to check *BAS1* complementation | ggacgttgaagggctcaa |
| SAT1 before excision R |  | ggtacagatggtactagacaaaaatatcaaaccaatca |
| BAS1-1300-F |  | ggaccaagcctgctagtactaacg |
| ARG4 internal R |  | TGATTTAGCTGATTATTTAGTTAGA |
